# The Danish High Risk and Resilience Study – VIA 7 - a cohort study of 520 7-year-old children born of parents diagnosed with either schizophrenia, bipolar disorder or neither of these two mental disorders

**DOI:** 10.1186/s12888-015-0616-5

**Published:** 2015-10-02

**Authors:** Anne A. E. Thorup, Jens Richardt Jepsen, Ditte Vestbjerg Ellersgaard, Birgitte Klee Burton, Camilla Jerlang Christiani, Nicoline Hemager, Mette Skjærbæk, Anne Ranning, Katrine Søborg Spang, Ditte Lou Gantriis, Aja Neergaard Greve, Kate Kold Zahle, Ole Mors, Kerstin Jessica Plessen, Merete Nordentoft

**Affiliations:** 1 Mental Health Center Copenhagen, Research Unit, Mental Health Services, Capital Region of Denmark, University of Copenhagen, Copenhagen, Denmark; 2 Child and Adolescent Mental Health Center, Research Unit, Mental Health Services, Capital Region of Denmark, University of Copenhagen, Copenhagen, Denmark; 3 Research Department P, Risskov, Aarhus University Hospital, Copenhagen, Denmark; 4 The Lundbeck Foundation Initiative for Integrative Psychiatric Research (iPSYCH), Aarhus, Denmark; 5 Center for Neuropsychiatric Schizophrenia Research, Psychiatric Center Glostrup, Mental Health Services Capital Region of Denmark, University of Copenhagen, Copenhagen, Denmark

**Keywords:** Genes, Environment, Neurocognition, Developmental psychopathology, Early signs, Interdisciplinary

## Abstract

**Background:**

Severe mental illnesses like schizophrenia and bipolar disorder are known to be diseases that to some extent, but not entirely can be understood genetically. The dominating hypothesis is that these disorders should be understood in a neurodevelopmental perspective where genes and environment as well as gene-environment-interactions contribute to the risk of developing the disease. We aim to analyse the influences of genetic risk and environmental factors in a population of 520 7-year-old children with either 0, 1 or 2 parents diagnosed with schizophrenia spectrum psychosis or bipolar disorder on mental health and level of functioning. We hypothesize that a larger proportion of children growing up with an ill parent will display abnormal or delayed development, behavioural problems or psychiatric symptoms compared to the healthy controls.

**Methods/design:**

We are establishing a cohort of 520 7-year-old children and both their parents for a comprehensive investigation with main outcome measures being neurocognition, behaviour, psychopathology and neuromotor development of the child. Parents and children are examined with a comprehensive battery of instruments and are asked for genetic material (saliva or blood) for genetic analyses. The participants are recruited via Danish registers to ensure representativity. Data from registers concerning social status, birth complications, somatic illnesses and hospitalization are included in the database. Psychological and relational factors like emotional climate in the family, degree of stimulation and support in the home and attachment style are also investigated.

**Discussion:**

Data collection started January 1, 2013, and is successfully ongoing. By Aug 2015 424 families are included. About 20 % of the invited families decline to participate, equal for all groups.

## Introduction

Schizophrenia and bipolar disorder are among the most severe and disabling mental disorders, influencing both patients, relatives and society [1]. Our knowledge about the etiology and development of these diseases is still limited and therefore prevention strategies are not specific or proven effective.

It has recently been shown that mental diseases like schizophrenia, major depression and bipolar disorder may be quite closely related genetically, meaning that there is a considerable genetic overlap between the three different diagnoses [2]. Based on this recent meta-analysis it was found that offspring of parents with one of the mentioned diseases not only had an increased risk of developing the same illness as their ill parent, but also an increased risk of developing the other two disorders.

*Schizophrenia* is known as a severe and complex mental disorder, most likely caused by several interacting genetic and environmental factors, each having only a limited effect and predictive value. The dominating hypothesis is that schizophrenia is a neurodevelopmental disorder [3]. The genetic contribution to the etiology of schizophrenia is among the best investigated in psychiatry and is well-documented in family, twin and adoption studies as well as in GWAS studies [4]. Also, studies have shown that genetic risk factors and environmental factors may interact with and be influenced by each other and thus also influence the risk of developing psychosis or bipolar disorder [5].

Children born to parents with *bipolar disorder* also have an increased risk of developing other mental disorders and specifically early-onset bipolar spectrum disorders [6, 7]. Most adults with bipolar disorder experience their first mood symptoms before the age of 21 years, whereas most children at high genetic risk who develop bipolar disorder later in life show manifest symptoms of the disorder much earlier, often before the age of 12 years.

### Objectives

The aims of the Danish High Risk and Resilience Study-VIA 7 are:

1. to analyse the influences of familial risk and environmental factors, including rearing conditions in childhood among a population of 7-year-old children with either 0, 1 or 2 parents diagnosed with schizophrenia spectrum psychosis or bipolar disorder, especially focusing on psychopathology, cognition, neuromotor, and psychosocial development;
2. to identify early risk markers of schizophrenia and bipolar disorder to establish a basis for future primary preventive interventions in the premorbid phase;
3. to get an overview of the kind and the intensity of help and support that the families have received from the community from the time when the child was born until the age of 7.

## Background

### Optimal designs for investigating gene-environment interactions

#### The high risk approach

Theoretically, optimal designs for investigating interaction between environmental and genetic risk factors in childhood and adolescence with respect to development of schizophrenia or bipolar disorder, would be randomized clinical studies with the individuals being exposed to poor environmental conditions, or adoption or twin studies. However, randomized clinical studies are not ethically acceptable and adoption studies with monozygote twins growing up under different conditions, are very rare.

Early signs of psychopathology and development of schizophrenia or bipolar disorder are rarely found in the general population, which makes studies of populations with an increased risk profile (‘high risk studies’) highly relevant for investigating the development of these disorders and the influence of genetic risk. One of the most famous studies in the field of schizophrenia research is ‘The Copenhagen High Risk Project’ [8], which so far is one of the largest studies, covering 207 children of mothers diagnosed with schizophrenia. It was started in 1962, when the children were on average 15 years old, and the study has contributed to our current knowledge with considerable and important results. One of its major contributions was to show that schizophrenia was at least partly familial. It was also one of the first studies to show the specific impairments in these children at high risk in domains of e.g. neuromotor development, behaviour, cognition and emotional problems [9, 10]. Antipsychotic medication was not introduced until the early fifties [11], thus making it is likely that the majority of the parents who participated at that time were not treated with antipsychotics in the early years of the children’s lives. In other words, they may have been quite severely ill and impaired by their psychotic symptoms in the early years of the children’s lives.

However, much has happened since 1962 - both concerning fundamental genetic understanding, diagnostic procedures, methods for measuring neurocognition and in terms of treatment of the disease. The social understanding in society and the public attitude towards psychiatric patients have changed, although stigma and isolation are still major issues. More insight into the diverse psychopathology, the neurocognitive deficits and the psychosocial development of children who later experience an onset of schizophrenia has emerged. We therefore found it relevant and important to use this knowledge in the design of a new high risk study of about the same size and to start examining these children at an even earlier age. Further, it is relevant to use up-to-date, validated and reliable methods capturing the special aspects of development that are relevant for this group.

It lies outside the scope of this paper to review all existing familial high risk studies, although relevant. For the earliest studies, the so called first generation HR-studies a thorough review [12] reports a generalized tendency to find developmental delays and a variety of problems or difficulties that these children may experience already during middle childhood and adolescence. These include neurointegrative problems, social impairments, early syptomatology, and deficits in attention processing and neuromotor functions. Also the emerging evidence for the influence of family enviroment is underlined. A need for future work to clarify developmental patterns within the same individuals is emphasized. A later review from 2003 is in accordance with these early results [13]. However, in general, the field is characterized by different designs and several shortcomings, e.g. only children with an affected mother, children with an affected 1^st^ degree relative, few or no follow-ups, and widespread age groups sometimes with small number of participants. For example the Rochester Family Study [14], the Swedish High Risk Study [15] and the Emory University Project [16] all included only between 25 and 61 HR subjects. The Helsinki High Risk Study [17] included 204 schizophrenia HR children and 204 controls and performed follow-up after 15 years. The group behind the Boston National Collaborative Perinatal Project HR Study (BNCPP [18]) and the Harvard Adolescent High Risk Study [19] has published several papers recently including follow-ups. The Edinburgh High Risk Study has a slightly different approach since it includes children with 2 or more 1^st^ og 2^nd^ degree relatives and has done quite long follow-ups. Also the Israeli High Risk Study (IHRS [20, 21]) and the Jerusalem Infant Development Study (JIDS) [22] deserve mentioning for contributing to the current knowledge. All the studies are pointing in the same direction supporting the neurodevelopmental hypothesis, but no study has to our knowledge been made with all children having the same age, including genetic material in combination with a thorough estimate of environmental factors on a representative cohort.

More recently, research concerning bipolar disorder has started to look at the same patterns, although it has long been well known that a family history of bipolar disorder is the strongest known risk factor for developing the disorder. For example, it has been found that children with familial risk for developing bipolar disorder may show early signs of vulnerability [23]. This is seen as higher rates of anxiety, sleep and mood disorders in childhood and as an increased risk for developing substance abuse in adolescence. These children also show elevated levels of psychopathology and behavioral problems compared to children of non-bipolar parents [24]. There is evidence that those individuals who later develop bipolar disorder report early signs of illness long before adulthood [25]. As with schizophrenia a staging model has been proposed with four stages from well-being to ill, starting in the early ages of childhood with anxiety and sleep disorder, moving to adjustment problems and subaffective states before major depression, ending up in fullblown bipolar disorder [26]. Researchers in this field emphasize the need of a developmental perspective to understand more about the origin of this severe mental disorder and about possibilities for preventive interventions [27–29].

Several environmental factors in both the prenatal and the perinatal periods as well as through infancy and childhood are associated with the risk of developing schizophrenia later in life. This concerns factors regarding the physical environment, e.g. intrauterine infections, complications during pregnancy and birth, or growing up in an urban district [30, 31]. It is also relevant to study factors that influence the child’s psychological development, such as the psychosocial and emotional environment and the everyday surroundings of the child (e.g. growing up in an institution), the degree of illness severity and chronicity of the ill parent, the emotional climate in the family, and experiences of childhood trauma [13, 32, 33]. Offspring of parents suffering from a severe mental illness such as schizophrenia or bipolar disorder are at a higher risk of growing up under adverse circumstances and experiencing traumatic life events (including the possibility of losing contact with one or both parents), which may result in disturbances in attachment and in normal, early cognitive and emotional development [34]. Only few studies have explored the levels of the parents’ psychopathology and their ability to function as caregivers. The associations between the severity and course of illness of the parent, and the rearing circumstances of the child with neurological, cognitive, behavioural and psychopathological risk markers in early childhood have not been specifically investigated before.

#### Early markers of severe mental disorder

Individuals who later develop schizophrenia, show several characteristics during childhood on a group level. This includes lower IQ, cognitive deficits, delayed neuromotor development, language developmental difficulties, emotional problems and poorer social function in childhood as established in longitudinal high risk studies and birth cohort studies [13, 35–43]. Among the most frequently reported characteristics in children at genetic high risk is poor cognitive and neurological functioning. Due to their stability and the high correlation with the underlying genetic burden, these characteristics may be considered as ‘endophenotypes’ that reflect their specific underlying genetic vulnerability [13, 44, 45]. Cognitive dysfunction, poor affective control, and subtle psychosis-like symptoms (PLIKS) are predictors for later development of psychosis in children at genetic risk for schizophrenia [42, 46]. Also, minor physical anomalies (MPAs) have been shown to be associated with an increased risk of psychosis [47].

For patients with bipolar disorder the literature points out that these patients have more frequently had attention deficits and disruptive disorders, increased rates of misuse in adolescence, as well as increased rates of anxiety disorders before the onset of the disease [48]. From this longitudinal, prospective study with 141 offspring (age 12–21) of families with a proband with BP, the authors conclude that anxiety and externalising diagnoses predict major affective illness in adolescence. The presence of syndromal depression, ADHD, disruptive behavioural disorders, and cyclothymic hypersensitive temperamental traits are found to be risk factors for future development of bipolar disorder, although most subjects exhibiting these traits or states do not develop the disease [25].

## Method

### Settings

We will establish a cohort of 520 7-year-old children from Denmark with different genetic risk profiles of schizophrenia and bipolar disorder in a follow-up design.

### Participants

We will conduct a thorough examination of 520 children aged 7 with either 0, 1 or 2 parents diagnosed with schizophrenia spectrum psychosis, which we define as schizophrenia, delusional disorder or schizoaffective disorder (ICD 10-codes: F20, F22 and F25 or ICD 8-codes: 295, 297, 298.29, 298.39, 298.89, 298.99) or bipolar disorder (defined as ICD 10 code F31 or ICD 8-codes: 296.19, 296.39). All children must be born in Denmark to ensure that full information via register data from both parents is accessible; − this is necessary to limit stratification in the genetic analyses. Analyses combining data from the Danish CPR Register (Civil Registration System [49]) and the Danish Psychiatric Central Research Register [50] show that each year approximately 200 7-year-old children are from families where at least one parent is affected with schizophrenia or schizophrenia spectrum psychosis. The number of children with one parent with bipolar disorder is expected to be similar. Approximately 20–30 7-year-old children have *two* parents diagnosed with either schizophrenia or schizophrenia spectrum psychosis and/or bipolar disorder in Denmark every year. During an inclusion period of approximately three years, we will randomly select the following stratified sample of 520 7-year-old children from all parts of Denmark for examination:

- at least one parent, either a mother or a father, diagnosed and registered with a schizophrenia spectrum psychosis; *N* = 200.
- at least one parent, either a mother or a father, diagnosed and registered with bipolar disorder; *N* = 120.
- neither of the parents being treated or registered in mental health services as in- or outpatients for the above-mentioned diagnoses; *N* = 200.

Group (a), (b), and (c) will be matched on urbanicity, community, gender and exact age of the child. Among the children included in group (a) or (b) 30–40 children are expected to have *two* ill parents, and form a special subgroup within the final cohort. The reasons for including a group of low risk controls are to evaluate gene-environment interactions with a control group and to create Danish norms for some of the outcome measures. Finally, this makes it possible to keep the child’s risk status concealed for the child investigator, thus not introducing bias due to specific expectations towards the child.

#### Age seven

The reason for choosing age 7 is that most children in Denmark have started school at this age, which thus represents an important developmental step for the child. Starting school is characterized by increased demands on the child, cognitive and academic, as well as social and practical. From a neurocognitive perspective this phase of a child’s life is characterized by a very rapid cognitive development, e.g. in the frontal lobes as demonstrated in a metaanalysis [51]. At the same time the early development of the child is not too distant to be recalled for the parent being interviewed.

### Exposures

#### Genetic exposure, polygenic risk scores and family history

The Danish Neonatal Screening Bio-bank is a national bio bank holding dried blood spot-samples from all new-borns since 1981 collected for genetic testing for e. g. phenylketonuria [43, 48–50]. All children in VIA 7 are identified in the Danish Neonatal Screening Bio-bank, their DNA extracted, whole-genome amplified and genotyped on the Illumina PsychChip, which is a whole-genome SNP array developed in collaboration with the Psychiatric Genetics Consortium (PGC) for genome wide association studies (GWAS). The data is processed using the bioinformatics pipeline of PGC. Similarly, DNA from blood samples from their parents is genotyped on the same platform. Polygenic risk scores predict case–control status in GWASs of schizophrenia [52, 53]. Constructed from risk alleles at thousands of genetic loci, the polygenic risk scores configure a simple and robust technique to study at a population level an individual’s genetic risk for polygenic traits, e. g. schizophrenia or bipolar disorder. Here, we will test if polygenic risk score alone and in combination with family history is associated with antecedents of schizophrenia or bipolar disorder and any observed associations of environmental exposures. Furthermore, it is possible to use the DNA from the blood spots and from saliva samples at age seven for genome-wide studies of methylation status at birth compared to age seven using the Infinium HumanMethylation450 BeadChip Kit (Illumina). Thus in VIA 7 we will be able to compare the epigenetic blueprint at birth with status at age seven and at later follow-ups. This may allow us to identify signatures of environmental exposures.

#### Environmental exposures

We aim to study *biological, social and emotional* exposures during the prenatal, perinatal, and infant period and in childhood. The prenatal and perinatal conditions of interest are maternal use of medication, tobacco, drugs and alcohol during pregnancy: pregnancy and birth complications and subjective constraints/stressors in the mother’s life. Specific environmental predictors will be extracted from registers, e.g., parental age (CPR register), birth complications (Danish Medical Birth Register/Danish National Hospital Register) [54] or identified through interviews with the mother. The combination of informant-based information concerning their current and earlier health status and other relevant life events, together with the information derived from registers, will enable us to make a more consistent quantification of several important pre- and perinatal exposures and confounders during pregnancy and up to age seven.

The *biological exposures* which play a role during infancy and childhood are among others neuro infections, cranial trauma or other severe somatic diseases, and failure to thrive. This information will be extracted from the Danish National Hospital Register [55], and from interviews with the mother/caregiver.

The *social and emotional* exposures like growing up with a parent with a severe mental disorder, number of changes in address and in institution/kindergarten, removal from home, parents’ social and financial status, prenatal stress, early postnatal rearing conditions, divorce and death of a close relative etc. will be collected. Some of these exposures can be extracted from complete longitudinal national registers (Statistics Denmark, IDA database [56]), some will be obtained from interviews.

*Psychopathology as an exposure*: Both biological parents and other important adults living with the child will be examined with Present State Examination [57] to assess current and life time mental health status. If the parent has ever had any symptoms of depression, mania, psychosis or negative symptoms, the symptom level of the present month will be rated, too, (depression by Hamilton Depression Scale [58], mania by Young Mania Rating Scale [59] and psychotic symptoms by Schedule for Assessment of Positive Symptoms (SAPS [60]) and negative symptoms by Schedule for Assessment of Negative Symptoms (SANS [61]).

The *rearing environment* will be studied in different ways. The emotional climate surrounding the child is measured through an interaction test with caregiver and child and by home visits (see section 3.6 Instruments and outcomes and Table 1, 2, and 3 for details).

**Table 1:** Instruments used for the assessment of the neuromotor and cognitive domains of the 7-year-old children participating in the Danish High Risk and Resilience Study-VIA 7

Domains	Subdomains	Child	Caregiver	Teacher
Neuromotor and physical measures	Motor development and milestones	Movement ABC [84]	Anamnestic interview (always including biological mother if possible)	
	do	Height, weight, head circumference, arm span		
	Motor speed and dexterity	Finger tapping [82]		
		Grooved Pegboard [83]		
	Minor physical anomalies (MPAs)	Waldrop Scale [103], 3D photo		
Cognition	Verbal memory and visual memory	Word Selective Reminding and Memory for Stories from Tomal-2 [85], RCFT: Rey Complex Figure Test and Recognition Trial [86]		
	Attention	RVP (rapid visual information processing; 3-5-7 mode) from CANTAB [88]. Conners CPT (Continuous Performance Test [96])		
	Receptive and expressive language (incl. grammar and pragmatic aspects)	TROG-II (Test for Reception of Grammar, [93])		CCC (Children’s Communication Checklist-II [112])
	Speed of Processing	Verbal Fluency and Trails A/B from D-KEFS [91]		
		Symbol Search, and Coding subtest from WISC-IV [90]		
	Executive functions (planning and flexibility)	SOC (Stockings of Cambridge) and IED (Intra-Extra Dimensional Shift test) from CANTAB [88].		
	Executive functions (visual and verbal working memory)	SSP (Spatial Span) and SWM (Spatial Working Memory) from CANTAB [88] + Letter-number Sequencing and Arithmetic from WISC-IV [90].		
	Executive functions (error monitoring)	Flanker Task [97].		
	Social cognition	Revised Happe’s Strange Stories [100], Animated Triangles [101] and ERT (Emotion Recognition Task) from CANTAB [88]		
	Intelligence	RIST (Reynolds Intellectual Screening Test [92])		
	Visual selective attention and perceptual speed	Visual Attention Battery from University of Copenhagen [99]		
	Smell identification	B-SIT (Brief Smell Identification Test, [94])		
	Imagination/creativity	Pattern Meanings [95]		
	Decision making	CGT (Cambridge Gambling Task) from CANTAB [88]

**Table 2:** Instruments used for the assessment of the psychiatric, behavioral and environmental domains of the 7-year-old children participating in the Danish High Risk and Resilience Study-VIA 7

Domains	Outcomes	Child	Caregiver	Teacher
Emotional and psychiatric symptoms	Psychiatric symptoms, incl. depression, anxiety, psychotic symptoms, thought disorders, PLIKS (psychosis like symptoms), obsessive-compulsive symptoms, eating disorders, sleep disturbances, self harming behavior and traumatic life events	K-SADS-PL [68]	K-SADS-PL [68]	TRF (Teachers Rating Form of Child Behavior Checklist (CBCL [69])
			CBCL (Child Behavior Checklist [69])	
	Attention/hyperactivity		ADHD-Rating Scale [72]	ADHD-Rating Scale [72]
	Affect regulation/flexibility	CEMS (Children’s Emotion Management Scale, [80])	BRIEF (Behavior Rating Inventory of Executive Function [87])	BRIEF (Behavior Rating Inventory of Executive Function [87])
	Anxiety	STAIC (State-Trait Anxiety Inventory for Children [81])		
	Self-esteem and quality of life	Kidscreen-27 ([78] and ‘Sådan er jeg’ (‘This is how I am’ [113])		
	Stress	Hair test for cortisol		
	Subjective stress	Items from DLSS (Daily Life Stressor Scale [79])		
Behavior	Social functioning		SDQ (Strengths and Difficulties Questionnaire [75])	SDQ (Strengths and Difficulties Questionnaire [75])
	do		Vineland Adaptive Behavior Scales –II [77]	
	Autism spectrum traits		SRS (Social Responsiveness Scale [76])	SRS (Social Responsiveness Scale [76])
	Attachment constructs	SSAP (Story Stem Assessment Profile [108])	CHQ (Caregivers Helplessness Questionnaire [107])	
Environment and emotional climate	Stimulation and support in actual rearing environment	HOME Inventory [104]	HOME Inventory [104]	
	Interaction with caregiver	Tangram Puzzle [106]	Tangram Puzzle [106]	
	Perceived support from social network (adults)		SPS (Social Provision Scale [109])	
	Expressed emotions		FMSS (Five Minute Speech Sample [105])

**Table 3:** Instruments used for the assessment of the parents of the 7-year-old children participating in the Danish High Risk and Resilience Study-VIA 7

Domains	Ill parent	Other parent	Actual caregiver, if not parent
Mental health (life time)	SCAN (Schedule for Clinical Assessment in Neuropsychiatry [57])	SCAN (Schedule for Clinical Assessment in Neuropsychiatry [57])	SCAN (Schedules for Clinical Assessment in Neuropsychiatry [57])
Daily functioning	PSP (Personal and Social Performance Scale [114])	PSP (Personal and Social Performance Scale [114])	PSP (Personal and Social Performance Scale [114])
Actual state of illness	SANS (Scale for the Assessment of Negative Symptoms [61])	SANS (Scale for the Assessment of Negative Symptoms [61])	SANS (Scale for the Assessment of Negative Symptoms [61])
do	SAPS (Scale for the Assessment of Positive Symptoms [60])	SAPS (Scale for the Assessment of Positive Symptoms [60])	SAPS (Scale for the Assessment of Positive Symptoms [60])
do	Hamilton Rating Scale for Depression [58]	Hamilton Rating Scale for Depression [58]	Hamilton Rating Scale for Depression [58]
do	YMRS (Young Mania Rating Scale [59])	YMRS (Young Mania Rating Scale [59])	YMRS (Young Mania Rating Scale [59])
Affective regulation	ALS (Affective Liability Scale [115])	ALS (Affective Liability Scale [115])	ALS (Affective Liability Scale [115])
Intelligence	RIST (Reynolds Intellectual Screening Test [92])	RIST (part of Reynolds Intellectual Screening Test [92])	RIST (Reynolds Intellectual Screening Test [92])
Verbal working memory and speed of processing	Letter-Number sequencing and coding from WAIS [116]	Letter-Number sequencing and coding from WAIS [116]	Letter-Number sequencing and coding from WAIS [116]
Executive functioning, flexibility, risk taking	IED (Intra/Extra Dimensional Set Shift test), SWM (Spatial Working Memory), CGT (Cambridge Gambling Task) and RVP (rapid visual information processing; 3-5-7 mode) from CANTAB [88]		
Smell identification	B-SIT (Brief Smell Identification Test [94])		
Verbal fluency	D-KEFS Verbal fluency [91]		
Social cognition	AT (Animated Triangles [101]), TASIT-R (The Awareness of Social Inference Test – Revised [117]), ERT (Emotion Recognition Task) from CANTAB [88]		
Social functioning	SPS (Social Provision Scale [109])	SPS (Social Provision Scale [109]) PAM (Psychosis Attachment Measurement [118])	SPS (Social Provision Scale [109]) PAM (Psychosis Attacment Measurement [118])
	PAM (Psychosis Attachment Measurement [118])		
Relation to child			CHQ Caregivers Helplessness Questionnaire [107]
Expressed emotions			FMSS (Five minute speech sample)
Interaction with child			Tangram Interaction test [106]

### Registers and bio-banks

A) The Danish Civil Registration System [49] was established in 1968. All persons alive and living in Denmark are registered and assigned a 10 digit unique and personal identification number. Furthermore, data on gender, date of birth, place of birth, continuously updated information on vital status (e.g. date of death and migration out of Denmark), and identity of parents, siblings, children and spouses are recorded, as are the addresses of each individual from 1972 and onwards.
B) The Danish Psychiatric Central Research Register: Data on psychiatric admissions in Denmark have been collected systematically since 1938. From April 1, 1969 these data were computerized and include all admissions to psychiatric hospitals. From January 1, 1995 outpatient contacts were included [50].
C) The Danish Medical Birth Register [54] covers information on all births in Denmark since 1973. It contains information on foetal presentation, earlier pregnancies, delivery, length, birth weight and weeks of gestation. It is now included as a part of The National Hospital Register [55], which was established in 1978 and information about all admissions to public hospitals in Denmark was prospectively recorded. Since 1995, outpatient visits were also registered.
D) The IDA Database [56], a Danish acronym for Integrated Database for Labour Market Research, contains longitudinal information on labour market conditions, automatically extracted from official records for all persons in the population, and their socio-demographic data.
E) The Children Register (http://esundhed.dk/sundhedsregistre/BDB/Sider/BDB.aspx), Statistics Denmark contains information about children whose parents lost custody of them and supportive interventions from the local authorities.
F) On the basis of the samples from the Danish Neonatal Screening Bio-bank, DNSB, it has been documented that whole genome amplification of DNA can be performed, and that fine mapping of candidate genes as well as genome-wide association analysis are possible [62, 63]. Furthermore, specific antibodies against infectious agents as well as a number of cytokines and inflammatory markers can be measured [64, 65]. This will enable us to make an exact measurement of several important exposures and confounders, direct measurement of genetic markers of relevance for infections or schizophrenia/bipolar affective disorders compared to data on family history, and will dramatically change the precision and interpretability in comparison to most studies currently available.

### Instruments and outcomes

The children will be assessed with a number of instruments to evaluate neuromotor and neuro- and social cognitive functions, behaviour and psychiatric symptoms [66, 67]. All outcome measures will be examined with validated instruments, specifically developed and selected for this young age group, sensitive to small changes and suitable for later follow-up, if relevant (see Tables 1 and 2).

The main outcomes and how they are captured are listed below:

- *different psychiatric and emotional symptoms*: anxiety, depression and obsessive symptoms, attention deficits and conduct disorders, psychotic and attenuated psychotic symptoms from K-SADS-PL (Schedule for Affective Disorders and Schizophrenia for School-Age Children [68]) and from CBCL (Child Behaviour Checklist [69, 70]), ADHD-RS (Attention Deficit Hyperactivity Disorder Rating Scale, Du Paul [71, 72]. PLIKS (Psychosis like symptoms) are covered by the psychosis supplement from K-SADS-PL, which is done with all children; no matter what they answer in the screening interview (cut-off is not used). A special scale, the SIPS scale [73], is used to register the milder, doubtful or subtle symptoms, e.g. rare and very brief experiences of mild auditory hallucinations like hearing someone calling his or her name etc. The tester will fulfil the TOF (Test Observation Form [74]) to include the clinical impression and performance during testing.
- *social function/behaviour:* SDQ (Strength and Difficulties Questionnaire [75], SRS (Social Responsiveness Scale [76], Danish Version, Hogrefe, Psykologisk Forlag, Copenhagen) and Vineland Adaptive Behavior Scale II, (only the subscale concerning social behaviour [77] is given to the parents). Questionnaires concerning the child’s own experiences and subjective viewpoints on self-esteem (‘Sådan er jeg’ in Danish meaning ‘This is me’), quality of life (Kidscreen, Danish Version, Hogrefe, Psykologisk Forlag, Copenhagen 2011, [78]), perceived stress (items from DLSS (Daily Life Stressor Scale [79]) and affect regulation/emotional control via selected single items from the questionnaire CEMS (Children’s Emotion Management Scales [80]) and STAIC [81].
- *fine motor speed and dexterity* (Finger Tapping Test [82] and Grooved Pegboard [83]) and gross motor skills (Movement Assessment Battery for Children-2: Movement ABC −2 [84]).
- *cognitive function*: aspects of verbal memory (Test of Memory and Learning, 2nd Edition: TOMAL-2 [85]) and visual memory (Rex Complex Figure Test: RCFT [86]), executive functioning (Behavior Rating Inventory of Executive Function, BRIEF [87]), subtests of CANTAB (ERT: emotion recognition task, RVP: rapid visual information processing, SWM: Spatial Working Memory, SSP: Spatial Span, SOC: Stockings of Cambridge, IED: Intra-Extra Dimensional Shift test, CGT: Cambridge Gambling Task [88, 89]), subtests from WISC-IV [90] and D-KEFS (Delis-Kaplan Executive Function System [91]), intelligence: Reynolds Intellectual Screening Test: RIST ([92] Danish Version, Hogrefe, Psykologisk Forlag, Copenhagen, 2011), impressive language (Test for Reception of Grammar TROG-2 [93], smell identification (B-SIT [94]) and creativity (Pattern Meanings [95]).
- *aspects of attention, error monitoring/early information processing*: Conners Continuous Performance Test (Conners CPT [96] and Flanker Test [97, 98], which is a modified Eriksen-flanker paradigm implemented in E-prime 2 (Psychology Software Tools, Pittsburgh, USA). Test for Visual Attention [99] and aspects of social cognition (Emotion Recognition Task (ERT) from CANTAB (Cambridge Neuropsychological Automated Test Battery [88]), Happe’s Strange Stories [100] and Animated Triangles [101, 102]. For details, see Tables 1 and 2.
- A hair-sample will be used for cortisol analysis, indicating *level of stress* of the child during the previous three months, combined with interview data. This part of the study is administered as part of the Gene-Environment study and is optional.
- 3D photos of the children and the affected parent will be taken for investigation of facial dysmorphism. Examination of *minor physical anomalies* (MPAs) is performed using the Waldrop Scale [103].
- investigation of *the emotional climate and the home environment* in the families will take place in the home using the HOME instrument (Home Observation for Measurement of the Environment [104]). This semi-structured interview focuses on the level of support and stimulation in the child’s home environment combined with direct observations. A brief measure of the level of expressed emotions is obtained from FMSS (Five Minute Speech Sample [105]) and child–parent interaction is assessed by means of an observation study of Tangram Puzzle test [106]. The Tangram test is a test where the child is asked to make a (very) difficult puzzle within 5 min. The actual caregiver sits next to the child for support and is told that the test is meant to reveal the child’s cognitive skills. The interaction between the adult and the child is videotaped and later coded from a manual). The actual caregiver will be asked about her or his degree of feeling helpless or out of control in relation to the child by means of the Caregiver Helplessness Questionnaire (CHQ [107]). The Story Stem Assessment Profile [108] is suitable for age 7 for measuring attachment style constructs of the child. Also the parents’ perception of perceived support from their social network will be included in a questionnaire [109].
- *exposure to illness/distress*: The parents (and the actual caregiver if not the parent) will be interviewed with SCAN (Schedules for Clinical Assessment in Neuropsychiatry [57]). Also stepparents who have lived more than 12 months with the child will be interviewed if they are willing to participate. A timeline going 8 years back in time (from pregnancy until now) will be used to register the periods of mental problems of the parents during the child’s life. The mother will be interviewed in detail about pregnancy, and early development (medication, alcohol, tobacco, adverse life events, early regulation difficulties, milestones etc.). Data on the actual socio-economic status will be collected from the interview and drawn from registers.
- *adverse life events/childhood trauma:* In the anamnestic interview we ask the actual caregiver about any adverse life events that may have happened to the child. In the K-SADS-PL all questions about experiences that could lead to PTSD are asked; they include all kinds of stressing life events and trauma, and they are registered, even if there is no PTSD.

### Procedures

Seven Ph.D. students, three clinical assistants, three research nurses and two postdocs are responsible for carrying out the clinical characterisation of the children and the parents. The primary research nurse coordinates administrative issues concerning the contacting of the invited families, registers etc. A letter with a small folder briefly describing the investigation is sent to the child’s address. This is followed up by a phone call from the research nurse a few days later, inviting the family to a meeting to learn more about what the implications of participation are. Both parents are asked for written, informed consent before data collection can start. In case of divorced parents, where both parents have custody of the child, as is most common in Denmark, both parents have to accept the child’s participation.

The genetic study ‘Gene Environment’ is presented as a separate study that is optional for all participants and has its own written consent form. The genotypic information about the children is based on blood samples from the Danish Neonatal Screening Bio-bank and for the parents from whole blood or saliva samples, if consent is given. These will be extracted at the time of the examination of the children. Moreover, we also ask for saliva samples from the children for epigenetic analyses. However, we decided not to ask for fresh blood samples from the 7-year-olds for psychological reasons.

Two researchers are assigned to each family, one testing the child while the other (simultaneously, if possible) conducts the interview with the primary caregiver. At least one home visit in the family’s home is arranged if the family accepts it. A very high degree of flexibility is required when arranging the interviews to ensure high response rates. That means that the interviews can take place wherever the family prefers them to take place. All interviews are made face to face (not by telephone or Skype). Best estimate diagnoses from the K-SADS-PL interview are established at weekly conferences in the research group with a specialist or professor in child- and adolescent psychiatry present. Regular monthly meetings are arranged for the entire group to measure inter-rater reliability and to ensure that all instructions and manuals are used correctly by all group members. We will measure reliability of diagnoses and choice of supplement by having all interviewers score ten K-SADS-PL interviews (screening part) independently from each other.

All researchers are trained in the entire test battery and can thus substitute for each other if necessary, but continuity in the contact to the families is aimed for. The researchers are blinded to high risk or control status of the child to ensure objectivity in the assessment and for ethical reasons, since some children may not be aware that they have a parent who at some point had or currently has a mental disease. The primary caregiver is asked about the child’s development and actual status, and all adults are interviewed about their own mental health status and history. The interviews and the tests will last approximately 7–8 hours (not including breaks and meals) and are therefore spread over more than one day, typically in 3–4 meetings of approx. 3 hours. The parents and the child are offered compensation for taking the time to participate by way of a gift card and transportation is paid for. If the parents give permission, information from the child’s primary teacher will also be obtained, see Tables 1 and 2. All parents with a diagnosis of schizophrenia or bipolar disorder are asked to perform the large cognitive battery, while the other parent does the small cognitive assessment. In the control families one parent is chosen to perform the larger cognitive battery, based on matching with a schizophrenia high-risk family in terms of child age and urbanicity, while the other parent does the small cognitive battery. So, in all families one parent is tested with the larger cognitive battery and one is tested with the smaller battery (see also Table 3).

Under Danish law, we as professionals have an extended responsibility to provide help if needed. On the other hand, since it has been made clear to all families from the beginning, that this is not an intervention study, this may on a few occasions lead to an ethical dilemma. We are aware of that in a few cases we feel a need to give the family advice or make a written referral in order to get the family some kind of help for themselves or their child.

### Ethical approval and Danish Data Protection Agency

We asked the Danish National Committee on Health Research Ethics for approval of the main study – VIA 7. However, under Danish law a study like this that does not involve any kind of intervention or genetic material, does not need an ethical approval. The Gene-Environment part of the study obtained Ethical Approval at the outset of the study and The Danish High Risk and Resilience Study –VIA 7 was later incorporated into their protocol as an appendix, which has then been approved by the committee.

The project is approved by the Danish Data Protection Agency and follows all laws concerning the processing of personal data. We received permission to draw data from registers from the Danish Ministry of Health.

### Statistics

Analyses will be carried out using SPSS, statistical package 22.0 for Windows (SPSS Inc., Chicago, IL, USA). All children will be divided into three main diagnostic groups HR-sz (High Risk schizophrenia), HR-bip (High Risk bipolar) and CTR (controls) based on ICD-10 diagnoses of the parents from registers. We will carry out descriptive analyses using standard statistical methods (Pearson chi-square test, one-way ANOVA analyses, Mann–Whitney U-tests whenever appropriate) investigating differences in demographic characteristics, psychiatric history, and phenotypic presentation. Linear and logistic regression models will be used to adjust for confounding factors and to investigate mediating and moderating factors.

### Status

Data collection started on 1 January, 2013 and is going on successfully, because a majority of the invited families accept to participate. By 1 August 2015, 424 families have accepted to participate, and 83 have declined, including some families that we were unable to contact since telephone numbers could not be found. Those who decline to participate are equally distributed between all three groups. Almost all included families fulfil the comprehensive data battery. Only eight families withdrew their consent after inclusion until July 2015. More than 80 % of the participants of the VIA7 study also accept to participate in the Gene-Environment Study by donating genetic material.

## Discussion

This section will briefly discuss the advantages and the limitations of our study. Also, we will mention a few considerations about how this study fits into the concept of developmental psychology and the newly defined and still developing project RDoC (NIMH Research Domain Criteria) that outlines new principles for how to capture and understand mental illness in a more dimensional way, not limited by traditional diagnostic boundaries.

### Strengths

- This study is to our knowledge the first study to include such detailed information about the environment of the child, including the thorough description of the child’s development and symptoms during the first 7 years of life.
- Our study is unique in that all children in the VIA 7 cohort have nearly the same age (within 12 months). This gives us a very detailed level of information of this exact age group, and also makes all comparisons much more accurate.
- Further, this population based selection of cases and controls at same age limits the heterogeneity of data and will thus increase power, e.g. with respect to environmental exposures. Data from Danish Registers allow us to invite a more representative sample of all high-risk children, since also parents, who are not actively attending treatment, are invited.
- Our non-biased sampling of exposures supplemented by detailed interviews will increase the validity of exposures.
- The very comprehensive mapping of cognition, psychopathology, MPA’s etc. will allow a thorough description of multiple important domains of child development.
- The interdisciplinary approach with data spanning from genetics and biometry to home environment and attachment measures is unique and allows integrative analyses and multidisciplinary interpretation.
- Participation rates are very high and drop-out rates are negligible in spite of the time consuming battery.
- DNA material from the children are collected both at birth and at age seven and will allow us to study epigenetic changes over time and comprehensive genotyping of risk alleles.
- The identification of biological signatures of exposures in utero as measured at birth is absolutely unique.
- With a planned follow-up at age 11 we will be the first ever to examine a large sample of HR-children twice before adolescence, and we will be able to describe developmental trajectories in more detail than any other study.

### Limitations

Although participation rates are high, representativity and selection bias have to be considered before drawing any general conclusions from the results. About one fifth of all invited families do not have a telephone number that can be found online, which prevents us from getting in contact with them. We could speculate that these are the families with the most severe courses and most troubled lives. But the situation might also be the reverse, i.e. families where a diagnosis of severe mental illness belongs to the past and no longer influences their lives.

The clinical diagnoses from the registers are not always confirmed at the clinical research interview, leaving us in doubt whether this person really did suffer from e.g. schizophrenia at some point in the past, or whether the register-based diagnosis was in fact wrong. We may need to ask for permission to read all hospital records in those cases to confirm the diagnosis.

This study is not meant to be an intervention study. But from the feedback we already have received from some of the participating families, it is clear that attending the interviews, telling their life history and reviewing their child’s developmental history may in some cases reveal problems that need to be solved, or it may inspire the families to introduce changes in their ways of living. This will only affect the baseline data to a very limited extent, but must be kept in mind at all follow-ups.

### Perspectives

The perspectives for the VIA 7 study of children at age 7 are threefold.

First of all, we wish to characterize children at familial risk with a wide range of clinical measures and a comprehensive neurocognitive battery to explore their characteristics as a group and to identify endophenotypes at age 7. This is relevant when planning health services specially tailored for this group of vulnerable and often overlooked children and their families. These families are often struggling on two fronts: with the challenges of normal family life and with a severe mental illness at the same time. We will be able to identify and characterize those children who already at age 7 show the first signs of neurodevelopmental delay or abnormal discourse. This will enable us to establish interventions and develop better strategies to detect and relieve these difficulties for children in a similar situation in the future and maybe also to prevent further development into severe mental illness.

Secondly, the long term perspective of the study is to make follow-ups at age 11, 15 and 21 and further on for the next decades, if possible, and thus learn more about the children’s cognitive, emotional and social developmental trajectories on a group level. A follow-up study at age 11, called VIA 11, is already being planned and financial support has been obtained. The follow-up will include many of the same measures as in the VIA 7 but also brain imaging (functional and MRI), which will provide very important information about the development of the brain in relation to mental health and environment. Having two measure points before puberty is unique to this study. At present we do not know if the delays found at age 7 will level out at early puberty or if they will progress over time to even lower levels.

Finally, we aim to answer the question: who among these children will in time develop a severe mental disorder, either the one, that they are predisposed for or another mental disorder? And who will remain well? The follow-up data will be able to provide important information about protective factors, resilience and predictors of mental health status in adolescence.

### RDoC project

The design and the purpose of this study is entirely aligned with the ideas that form the basis of the NIHM’s RDoC Project [110]. The overall goal of the RDoC is to develop a new classification of psychopathology based on dimensions of neurobiology and behavioural measures. The basic principles are that mental illnesses are disorders of brain circuits, that neuroscientific methods can identify brain dysfunctions, and that knowledge about disordered circuits will show the way for future classification, assessment, and treatment. RDoC aims to inspire research that focuses on dysfunction across domains and levels of analysis. The domains that are included at the moment are: *negative valence, positive valence, cognitive systems, system for social processes, and arousal/modulatory systems.* Levels of analysis could be genes, molecules, cells, circuits, physiology, behaviour and self-report. Two other dimensions that are crucial to the RDoC project are developmental trajectories and environmental effects. They often interact and should be considered as integral parts of the structure.

The RDoC project is at its beginning, aiming to build a database that will change the understanding of psychopathology fundamentally. It has a new perspective on research, cutting across diagnostic categories, a perspective that is both useful and necessary when studying developmental psychopathology and early signs of mental illness [111]. The goals of this high risk study are to our conviction nicely aligned with the RDoC initiative in two fundamental ways: First, we are interested in the continuum of psychotic disorders and their expression. While we are collecting clinical information regarding diagnosis, this will help our data be compared to previous studies using DSM or ICD diagnoses. Nevertheless, we believe that the continuum of psychosis and its risk will be very well explored by our design. Second, the phenotypes we assess, whether behaviour, cognition, and so on, are continuous dimensions relevant to all psychotic disorders. While they were not chosen explicitly using RDoC domains, we are covering in some detail, some of those domains, such as working memory, social cognition, or negative affect and reward systems and thus our data will be informative within an RDoC framework. For example, the domain of negative valence/psychopathology is captured in the study both as categorical data (the diagnoses given on the basis of the K-SADS-PL interview) and as dimensional measures on the CBCL (Child Behavior Checklis [69]) and TRF (Teachers Rating Form [69]). The first gives us dichotomous data (diagnosis/no diagnosis), while the other two are continual scores based on information from both the actual caregiver’s and the actual teacher’s concerning observed behaviour and problems of the child and indicating child psychiatric problems. Further, this will be combined with the ratings of the clinical impression of the child during the clinical testing (TOF; Test Observation Form [74]) and will be followed up upon in VIA 11. In terms of the RDoC domain called ‘cognitive systems’ we have data covering all the subdomains (attention, perception, declarative memory, language behaviour, cognitive control and working memory). All together this approach to psychopathology is much in line with the developmental approach that the RDoC is based on. Further, with the perspective of adding scans and blood tests at the 11-year follow-up will give us information that fits into what RDoC calls ‘cells’, ‘molecules’ and ‘circuits’.

## Conclusion

This paper describes ’The Danish High Risk and Resilience Study – VIA 7’ a cohort study of 520 7-year-old children, who are born to parents with either schizophrenia, bipolar disorder or neither of the two diagnoses. We are aiming to capture both genetic factors and environmental factors. We investigate the children’s neuromotor, neurocognitive, and social and behavioral functioning as well as their psychiatric symptoms. We also include data from the parents, from teachers and data from Danish Registers.

It is quite unique that all children have the same age, that participants are selected in a population based manner and that we have two samples of genetic material from the children. A follow-up at age 11 is planned to ensure two measurements before puberty in all important domains, which is unique, too.
